# Association between hypertension and retinal vascular features in ultra-widefield fundus imaging

**DOI:** 10.1136/openhrt-2019-001124

**Published:** 2020-01-08

**Authors:** Gavin Robertson, Alan Fleming, Michelle Claire Williams, Emanuele Trucco, Nicola Quinn, Ruth Hogg, Gareth J McKay, Frank Kee, Ian Young, Enrico Pellegrini, David E Newby, Edwin J R van Beek, Tunde Peto, Baljean Dhillon, Jano van Hemert, Thomas J MacGillivray, on behalf of Northern Ireland Cohort of Longitudinal Ageing

**Affiliations:** 1 Research, Optos plc, Dunfermline, UK; 2 Centre for Cardiovascular Sciences, University of Edinburgh, Edinburgh, Lothian, UK; 3 The VAMPIRE Project, Computer Vision and Image Processing Group, School of Science and Engineering, University of Dundee, Dundee, Dundee, UK; 4 Centre for Public Health, Queen's University Belfast, Belfast, Belfast, UK; 5 Edinburgh Imaging Facility QMRI, University of Edinburgh, Edinburgh, UK; 6 The VAMPIRE Project, Centre for Clinical Brain Sciences, University of Edinburgh, Edinburgh, UK

**Keywords:** hypertension, imaging and diagnostics, microvascular disease

## Abstract

**Objective:**

Changes to the retinal vasculature are known to be associated with hypertension independently of traditional risk factors. We investigated whether measurements of retinal vascular calibre from ultra-widefield fundus imaging were associated with hypertensive status.

**Methods:**

We retrospectively collected and semiautomatically measured ultra-widefield retinal fundus images from a subset of participants enrolled in an ongoing population study of ageing, categorised as normotensive or hypertensive according to thresholds on systolic/diastolic blood pressure (140/90 mm Hg) measured in a clinical setting. Vascular calibre in the peripheral retina was measured to calculate the nasal–annular arteriole:venule ratio (NA-AVR), a novel combined parameter.

**Results:**

Left and right eyes were analysed from 440 participants (aged 50–59 years, mean age of 54.6±2.9 years, 247, 56.1% women), including 151 (34.3%) categorised as hypertensive. Arterioles were thinner and the NA-AVR was smaller in people with hypertension. The area under the receiver operating characteristic curve of NA-AVR for hypertensive status was 0.73 (95% CI 0.68 to 0.78) using measurements from left eyes, while for right eyes, it was 0.64 (95% CI 0.59 to 0.70), representing evidence of a statistically significant difference between the eyes (p=0.020).

**Conclusions:**

Semiautomated measurements of NA-AVR in ultra-widefield fundus imaging were associated with hypertension. With further development, this may help screen people attending routine eye health check-ups for high blood pressure. These individuals may then follow a care pathway for suspected hypertension. Our results showed differences between left and right eyes, highlighting the importance of investigating both eyes of a patient.

**Key questions**

**What is already known about this subject?**

- Changes to the retinal vasculature are associated with hypertension independently of traditional risk factors.

**What does this study add?**

- Semiautomated measurements of nasal–annular arteriole:venule ratio in ultra-widefield fundus imaging were associated with hypertension.
- Our results also showed differences between left and right eyes, highlighting the importance of investigating both eyes in a given patient.

**How might this impact on clinical practice?**

- With further development, this tool may help screen people attending routine eye health check-ups for high blood pressure. These individuals may then follow a care pathway for suspected hypertension. This could bring health benefits to the individual and economic benefits to society.

## Introduction

Hypertension is a common, generally asymptomatic condition that is a leading risk factor of death and disability worldwide.^1^ Around 30% of the adult population in the UK are thought to have hypertension,^2^ and approximately 30% of this group are estimated to be undiagnosed, leaving them at increased risk of cardiovascular disease.^3^

An image of the retina taken by a fundus camera or a scanning laser ophthalmoscope reveals a detailed view of the body’s microcirculation through non-invasive examination. Quantitative features derived from the retina, such as measurements of vascular calibre (ie, thickness of the blood column) near the optic disc, have been associated with increasing blood pressure^4–8^ and presence of hypertension.^9–13^ Typically, this association is characterised by a narrowing of the retinal arterioles, with little change to the venules. To mitigate for body size and eye magnification between individuals, the ratio of arteriole to venule calibre is often used as a surrogate for arteriolar calibre.^14^ For people attending routine eye check-ups, a test involving retinal imaging for undiagnosed hypertension would bring health benefits.

Analyses of the retina have been limited in part by the viewing field of imaging systems. For a 45° fundus camera, this is typically a circular area extending approximately 4 mm^15^ from the image centre. Ultra-widefield imaging with a scanning laser ophthalmoscope captures more of the retina in a single image^16^ and thus allows measurement of retinal vessel parameters in areas not previously investigated.

In this study, we assessed measurements of retinal vessels in ultra-widefield imaging for association with hypertension.

## Methods

The Northern Ireland Cohort for the Longitudinal Study of Ageing^17^ (NICOLA) is a long-term study of ageing in people aged 50 years and over with a projected 10-year follow-up. Participants were invited for a health assessment at the Wellcome Trust Northern Ireland Clinical Research Facility that included a review of cardiovascular, cognitive and respiratory function as well as visual health, including ultra-widefield imaging with a scanning laser ophthalmoscope (P200Tx, Optos, Dunfermline, UK).

**Figure 1:**
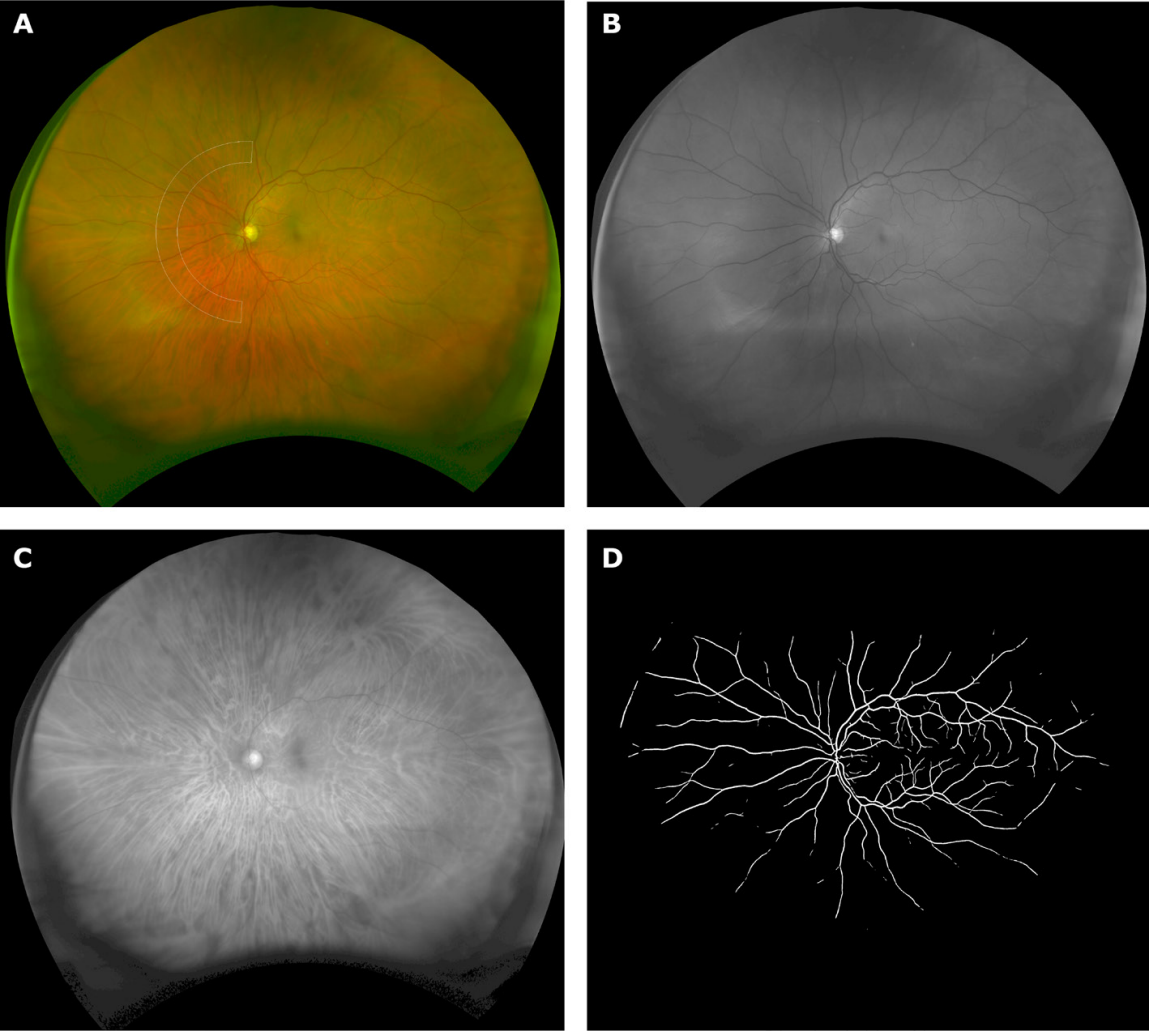
(A) Pseudo-colour image of the retina showing the region of interest, at a distance of 6.5–8.5 optic disc radii from the optic disc centre, in which nasal–annular arteriole:venule ratio was calculated. (B) Green laser (532 nm) image showing the retinal vasculature contrasted against the retinal surface. (C) Red laser (633 nm) image showing choroidal vessels as well as some of the larger surface venules. (D) Automatically generated retinal vascular map.

The ultra-widefield scanning laser ophthalmoscope generates simultaneous red and green images that are combined to give a pseudo-colour image of the retina (figure 1). Green light at a wavelength of 532 nm has a short penetration into the retina and is quickly absorbed by the blood column. Red light at 633 nm is preferentially absorbed by deoxygenated haemoglobin,^18^ causing venules to appear with greater contrast than arterioles. Each ultra-widefield image had dimensions of 4000×4000 pixels with an on-axis resolution of approximately 15 µm and represented a stereographic projection of the retinal surface. Pixel estimates of vascular calibre were converted to microns using a pixel to micron scale that varied with position in the image.^19^

Images and clinical data were extracted from the NICOLA study database. Inclusion criteria were people in the age range of 50–59 years, availability of scanning laser ophthalmoscope images in both eyes and availability of blood pressure readings. All participants who met these criteria at the time of database interrogation were included in the sample. Clinical systolic and diastolic blood pressure were derived from the average of two sitting measurements taken at rest in a quiet place, from the same arm, 1 min apart, using an automatic digital blood pressure monitor (Omron M10-IT) and arm cuff. Hypertension was defined as systolic blood pressure of ≥140 mm Hg and/or diastolic blood pressure of ≥90 mm Hg.

The nasal–annular arteriole:venule ratio (NA-AVR) is a novel morphometric parameter which extends the conventional definition of arteriole:venule ratio^20^ by taking vascular calibre measurements in a more peripheral region of the retina. Here, vessels are better separated and more distinct than near the optic disc where they frequently cross. NA-AVR was evaluated in each ultra-widefield image as follows:

1. The optic disc location, its approximate outline and the fovea location were manually annotated by a trained operator (author GR, who has greater than 7 years’ experience analysing retinal images). These landmarks were used to establish the zone of measurement as an annular segment subtending 180° nasal to the optic disc and fixed on a line passing through the centre of the fovea and optic disc. It extended from 6.5 to 8.5 optic disc radii from the optic disc centre (figure 1A). If the retina in either eye was obscured by eyelid, lash or other artefact within the zone of measurement, then the person was excluded from the study.
2. Next, automatic segmentation of the retinal vessels was performed using a validated computer algorithm described elsewhere.^21^ In brief, the green image was processed using scaled filters to increase vessel contrast with respect to the background. The filters exploited the morphology of the vessels as well as their cross-sectional intensity profile. A supervised machine learning classifier trained on a separate image set categorised image pixels as vessel or non-vessel (figure 1D).
3. The retinal vessels detected in the zone of measurement were overlaid on the original image. The operator modified the detected vasculature to correct for false-positive and false-negative detections and labelled each vessel segment as arteriole or venule according to their appearance and connectivity.
4. The calibre (in microns) for each vessel segment in the measurement zone was estimated as the average distance between parallel splines automatically fitted to the detected vessel edges.^22^
5. Finally, NA-AVR was calculated as the ratio of the average calibre of the three widest arterioles and the average of the three widest venules. Fewer arteriole and venule segments were used when less than three were available.

Intraobserver measurement repeatability for NA-AVR was assessed for the left and right eyes in a random subset (n=46) of the study cohort. Each operator interaction (above-mentioned steps 1 and 3) was repeated to generate a second set of NA-AVR measurements. To mitigate potential bias in the treatment of left and right eyes, 50% of the eyes chosen for reanalysis were mirrored in the vertical axis to simulate the appearance of the opposing eye. The operator was blinded to previous measurements, and the images were presented in a different order.

The operator was blind to all clinical information, such as sex and hypertensive status, during the stages of image measurement to mitigate for observer bias.

Two-tailed Student t-tests and the area under the receiver operating characteristic curve (AUC) were used to assess NA-AVR as a predictor for hypertensive status. Pearson’s linear correlation coefficient, R^2^, and a paired Student t-test were used to compare measures between eye pairs and in the repeatability analysis. The F-statistic was used to test significance for the relationship of NA-AVR with systolic and diastolic blood pressures. All CIs were evaluated at the 95% level.

Image processing and statistical analysis were undertaken using MATLAB (r2014a, The MathWorks, USA).

## Results

In total, 460 participants in the NICOLA study (aged 50–59 years, mean age of 54.6±2.9 years, 259, 56.3% women) met the inclusion criteria. Of these, 20 (4.3%) were excluded due to the retina being obscured in the measurement zone, leaving 440 available for analysis. The number of participants categorised as hypertensive from their clinical blood pressure measurements was 151 (34.3%), of which 57 (37.7%) were female.

**Figure 2:**
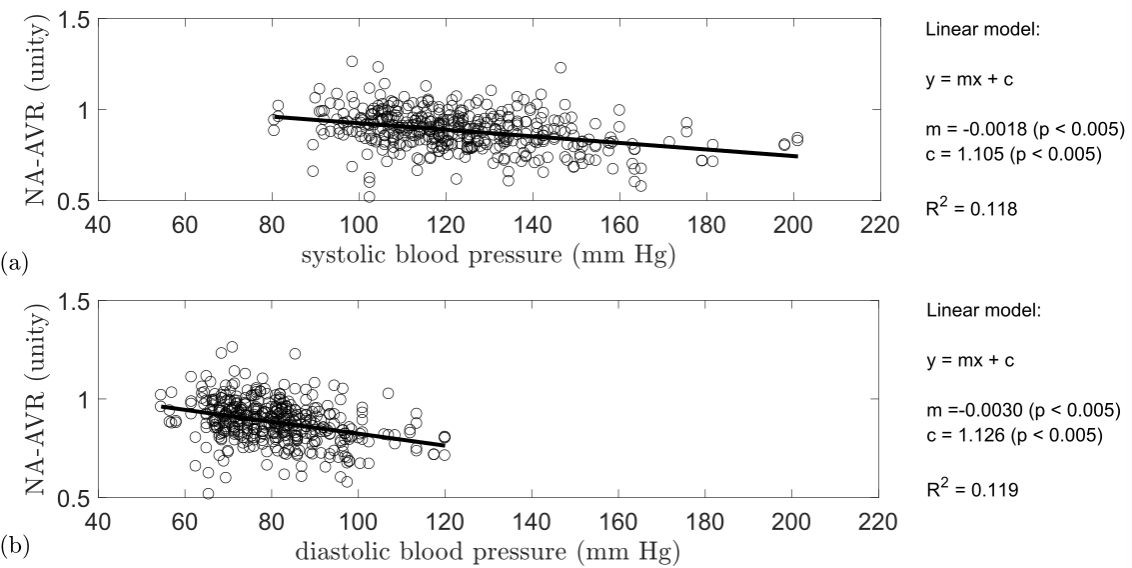
NA-AVR plotted against (A) systolic blood pressure (mm Hg) and (B) diastolic blood pressure (mm Hg). Also given is a linear model fitted to the data with corresponding R^2^ value. A reduction in NA-AVR was observed with increasing systolic and diastolic blood pressures. NA-AVR, nasal–annular arteriole:venule ratio.

A linear relationship between NA-AVR and blood pressure, both systolic and diastolic, was observed (figure 2). There was a reduction in NA-AVR with increasing systolic (R^2^=0.118, p<0.005) and diastolic (R^2^=0.119, p<0.005) blood pressures.

**Figure 3:**
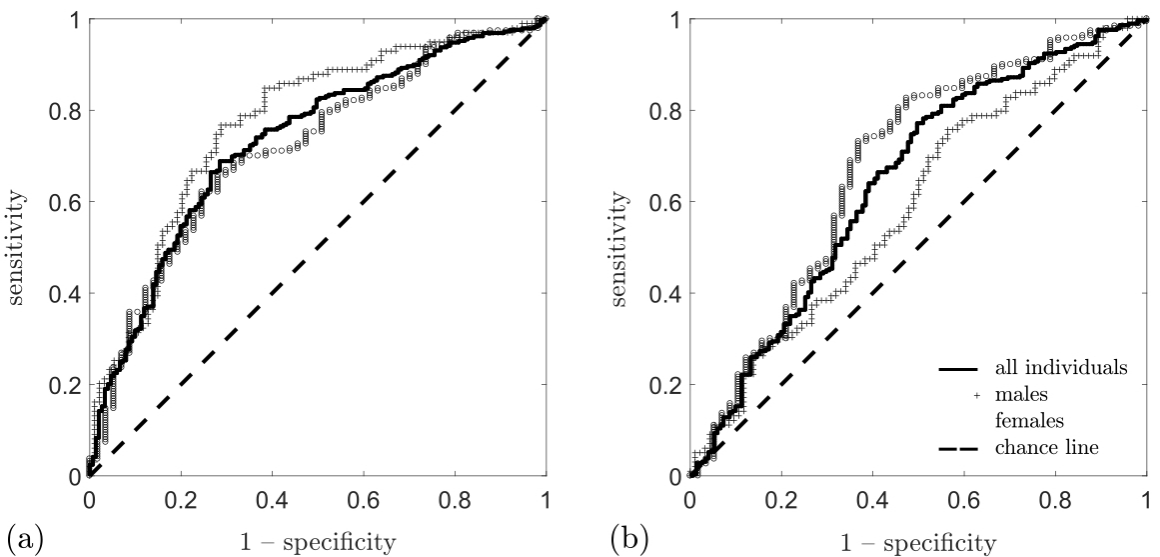
Receiver operating characteristic curve for nasal–annular arteriole:venule ratio as a detector of hypertensive status in the study cohort, showing sensitivity plotted against 1−specificity. Increased distance from the chance line indicates a better detector. Curves are displayed for all subjects (solid line), males (+) and females (○). (A) Using the left eye, the largest AUC was obtained for males, AUC=0.77. (B) Using the right eye, we obtained the largest AUC for women, AUC=0.68. AUC, area under the receiver operating characteristic curve.

Table 1 shows the AUC of NA-AVR for hypertensive status in the study cohort, with corresponding receiver operating characteristic curves in figure 3. For all participants, the AUCs were 0.73 (95% CI 0.68 to 0.78) in the left eye and 0.64 (95% CI 0.59 to 0.70) in the right eye, representing evidence of a statistically significant difference between the eyes (p=0.020). Further analysis by sex revealed that NA-AVR gave the best performance in the left eye for men, the AUC being 0.77 (95% CI 0.70 to 0.83) compared with 0.72 (95% CI 0.73 to 0.78) for women; this was not statistically significant (p=0.352). We determined that using the three widest arterioles and the three widest venules gave better performance than the use of one or five (see online supplementary table S2).

10.1136/openhrt-2019-001124.supp1 Supplementary data

**Table 1:** AUC for NA-AVR as a detector of hypertensive status in the study cohort

Measure	Group	Left eye		Right eye		P value
		AUC	CI	AUC	CI	
NA-AVR (unity)	All	0.73	0.68 to 0.78	0.64	0.59 to 0.70	**0.020**
	M	0.77	0.70 to 0.83	0.59	0.51 to 0.67	**<** **0.005**
	F	0.72	0.63 to 0.78	0.68	0.58 to 0.76	0.502

With 95% CIs and 5% significance level (p). Significant p values are in bold.

AUC, area under the receiver operating characteristic curve; F, female; M, male; NA-AVR, nasal–annular arteriole:venule ratio.

NA-AVR was lower in hypertension compared with normotension when stratified by both eye and sex (table 2). There was a difference in arteriolar calibre of 8.1 µm between people with normotension and hypertension (95% CI 6.0 to 10.2, p<0.005) but no evidence of a difference for venular calibre (0.6 µm, 95% CI −1.6 to 2.8, p=0.577).

**Table 2:** Mean (SD) NA-AVR stratified by eye and sex (all, M and F) in normotension and hypertension

Measure	Eye	Group	Normotension mean	n	Hypertension mean	n	P value	CI
NA-AVR (unity)	Left	All	0.90 (0.10)	289	0.82 (0.09)	151	**<** **0.005**	0.06 to 0.10
		M	0.91 (0.10)	99	0.82 (0.09)	94	**<** **0.005**	0.07 to 0.12
		F	0.89 (0.11)	190	0.81 (0.09)	57	**<** **0.005**	0.04 to 0.11
	Right	All	0.91 (0.09)	289	0.86 (0.11)	151	**<** **0.005**	0.03 to 0.07
		M	0.90 (0.11)	99	0.86 (0.11)	94	**0.024**	0.01 to 0.07
		F	0.92 (0.09)	190	0.86 (0.11)	57	**<** **0.005**	0.03 to 0.08
Arterioles (µm)	Left	All	86.6 (10.9)	289	78.5 (10.3)	151	**<** **0.005**	6.0 to 10.2
		M	87.3 (10.3)	99	79.0 (10.1)	94	**<** **0.005**	5.4 to 11.2
		F	86.2 (11.2)	190	77.7 (10.5)	57	**<** **0.005**	5.2 to 11.8
	Right	All	88.8 (10.2)	289	83.0 (10.2)	151	**<** **0.005**	3.8 to 7.8
		M	87.8 (11.0)	99	83.5 (9.6)	94	**<** **0.005**	1.4 to 7.3
		F	89.3 (9.7)	190	82.3 (11.0)	57	**<** **0.005**	4.0 to 10.0
Venules (µm)	Left	All	97.3 (11.1)	289	96.7 (11.1)	151	0.577	−1.6 to 2.8
		M	96.5 (10.3)	99	97.0 (10.8)	94	0.733	−3.5 to 2.5
		F	97.7 (11.5)	190	96.1 (11.5)	57	0.346	−1.8 to 5.1
	Right	All	98.1 (10.0)	289	97.0 (11.7)	151	0.295	−1.0 to 3.2
		M	98.3 (11.0)	99	97.5 (11.9)	94	0.624	−2.5 to 4.1
		F	98.0 (9.4)	190	96.1 (11.4)	57	0.212	−1.1 to 4.8

The mean diameters of the arteriolar and venular components (µm) used to calculate NA-AVR are also given. Two-tailed Student’s t-tests were used to test the null hypotheses of equal means at the 5% significance level (p) and to obtain 95% CIs for the differences between the means. Significant p values are in bold.

F, female; M, male; NA-AVR, nasal–annular arteriole:venule ratio.

**Figure 4:**
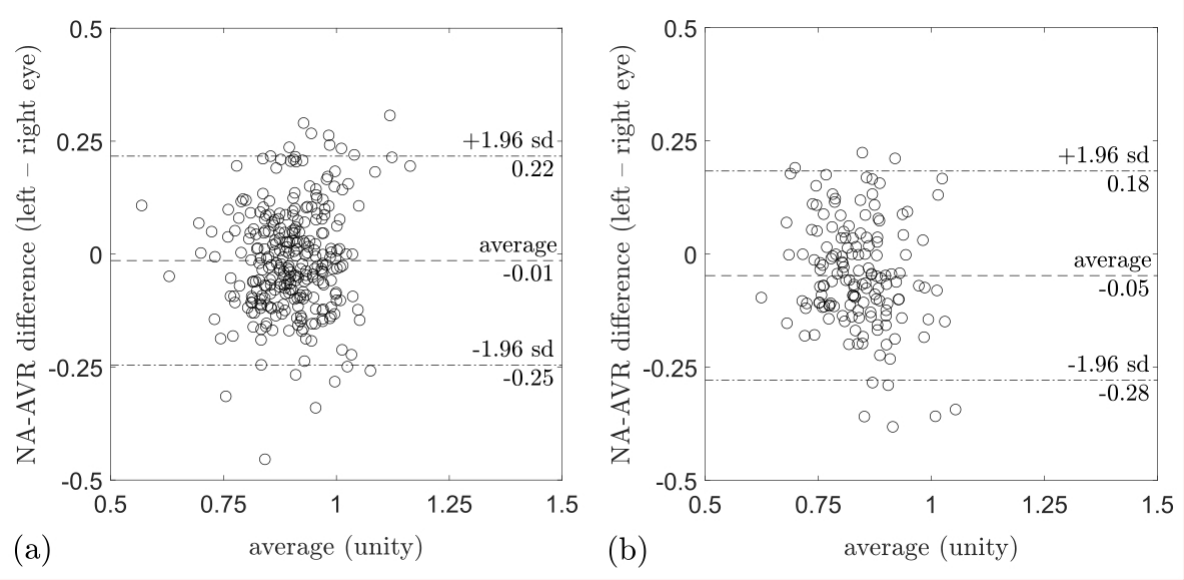
Bland-Altman plot for (A) NA-AVR in normotension and (B) in hypertension. The y-axis shows the difference in NA-AVR between left and right eyes, while the x-axis is the average of NA-AVR for each eye pair. In both normotension and hypertension, NA-AVR is smaller for the left eye compared with the right eye. The data points in (B) are further to the left compared with those in (A), indicating smaller NA-AVR in hypertension compared with normotension. NA-AVR, nasal–annular arteriole:venule ratio.

For all participants and subgroups by hypertension status, a weak correlation between the eyes was observed (table 3). Splitting NA-AVR into its component arteriolar and venular calibres revealed moderate correlations, which were R^2^=0.50 (95% CI 0.43 to 0.57) and 0.31 (95% CI 0.22 to 0.39) for arterioles and venules, respectively. NA-AVR measured in the left eye was smaller than that in the right eye, with a difference of −0.03 (95% CI −0.04 to −0.02, p<0.005; table 3). There was a left–right difference in arteriolar calibre of −3.1 µm (95% CI −4.1 to −2.0, p<0.005) but no evidence of a difference in venular calibre. Bland-Altman plots show the relationship for NA-AVR between eye pairs in subjects with normotension and hypertension (figure 4). A greater difference in NA-AVR between the eyes was observed in people with hypertension compared with those with normotension (0.03, 95% CI 0.01 to 0.06, p<0.005).

**Figure 5:**
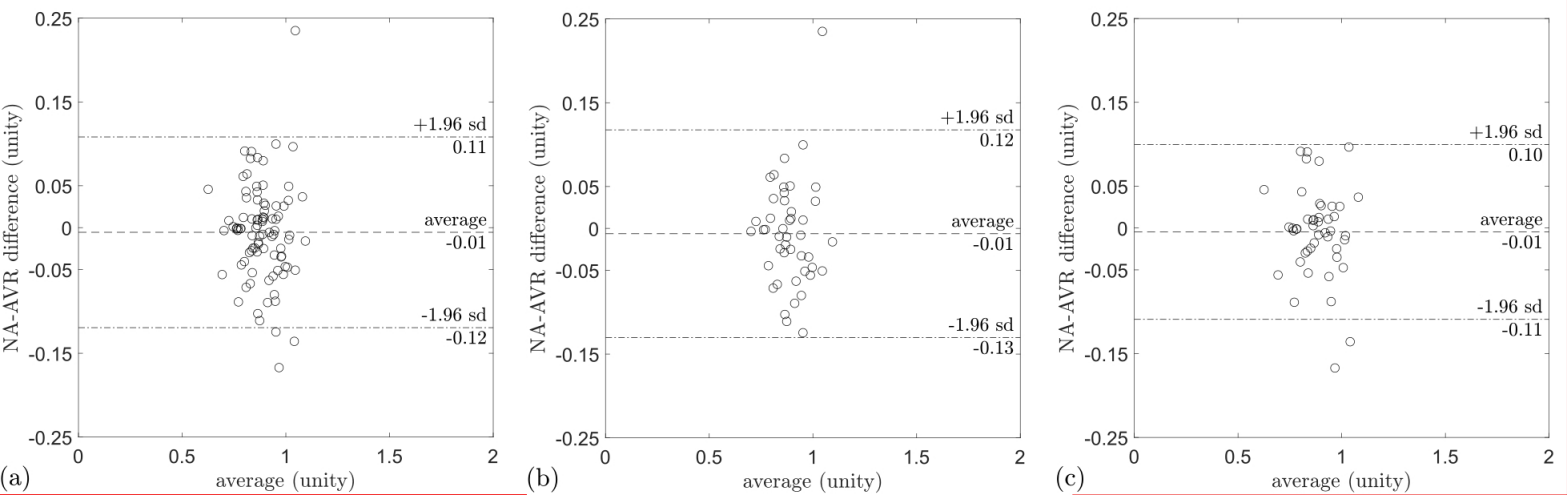
Bland-Altman plots for NA-AVR measurement repeatability, showing the difference between successive measurements (repeat−original) on the y-axis and the average of the two measurements on the x-axis for (A) all eyes, (B) left eyes and (C) right eyes. NA-AVR, nasal–annular arteriole:venule ratio.

**Table 3:** Pearson’s linear correlation coefficient (R^2^) and paired t-test results comparing NA-AVR between left and right eyes for groups: all subjects (all), HT and NT

Measure	Group	Pearson’s linear correlation			Paired t-test			N
		R^2^	CI	P value	Difference	CI	P value	
NA-AVR (unity)	All	0.34	0.26 to 0.42	<0.005	−0.03	−0.04 to −0.02	**<** **0.005**	440
	HT	0.29	0.14 to 0.43	<0.005	−0.05	−0.07 to −0.03	**<** **0.005**	151
	NT	0.30	0.19 to 0.40	<0.005	−0.02	−0.03 to −0.00	**0.040**	289
Arterioles (µm)	All	0.50	0.43 to 0.57	<0.005	−3.1	−4.1 to −2.0	**<** **0.005**	440
	HT	0.50	0.38 to 0.62	<0.005	−4.6	−6.2 to −2.9	**<** **0.005**	151
	NT	0.43	0.33 to 0.52	<0.005	−2.2	−3.5 to −0.9	**<** **0.005**	289
Venules (µm)	All	0.31	0.22 to 0.39	<0.005	−0.6	−1.8 to 0.6	0.317	440
	HT	0.43	0.29 to 0.55	<0.005	−0.3	−2.3 to 1.7	0.776	151
	NT	0.24	0.13 to 0.35	<0.005	−0.75	−2.2 to 0.7	0.311	289

The mean diameters of arteriolar and venular components (µm) used to calculate NA-AVR are also shown. With 95% CI and 5% significance level (p). Significant p values are in bold.

HT, hypertension; NA-AVR, nasal–annular arteriole:venule ratio; NT, normotension.

In the intraobserver analysis of 91 images, no evidence of significant differences was found in repeat measures of NA-AVR or arteriolar calibre. However, a small difference of 1.9 µm (95% CI 0.6 to 3.2, p<0.005) was observed between measurements of venular calibre (see figure 5, online supplementary figure S1 and table S1).

10.1136/openhrt-2019-001124.supp2 Supplementary data

## Discussion

NA-AVR and arteriolar calibre were reduced in hypertension compared with normotension, and there was a trend of decreasing NA-AVR with increasing systolic and diastolic blood pressures, which is consistent with previous literature.^23^

Differences were observed in arteriolar calibre and NA-AVR between left and right eye pairs in both normotension and hypertension. This differs from conclusions drawn in previous studies that measured vascular calibre closer to the optic disc. In the Rotterdam study, a subset of 100 subjects found no differences between eyes for arteriolar or venular calibre,^24^ while the Blue Mountains Eye Study (n=1546) concluded, based on between-eye correlations, that measurements from one eye could adequately represent the retinal vascular diameters of a particular person. However, in a subset of the data (n=576) where both eyes were graded by the same person, arteriolar calibre was significantly lower in the left eye, with no significant difference in venular calibre.^20^ This is in concordance with our findings. In other studies, it has been common practice to use only one eye per subject,^5–13^ usually the right eye, unless the quality was insufficient, in which case the left eye was used. This is presumably due to the assumption that differences in left and right eye morphology were insignificant. Recently, however, the assumption of symmetry in vessel morphology between the eyes has been questioned.^25^ For example, vascular branching and vessel tortuosity display asymmetry.^26^

NA-AVR for detection of hypertensive status differed between left and right eye pairs for men but not for women, where lower arteriole:venule ratios were found in men. The disparity between left and right eye performances with sex is a potentially interesting area for a future study. We do not currently know of any physiological reason for this effect.

This study had the following limitations. Blood pressure measurements taken in a clinical setting can suffer from the so-called ‘white-coat effect’ (at a rate of 9.4%–24.2%) where elevated measurements are recorded, resulting in overdiagnosis of hypertension, and masked hypertension (8.6%–17.0%) where lower measurements are recorded, resulting in underdiagnosis.^27^ This limitation of single clinical blood pressure measurements has led to the recommendation of 24 hours of ambulatory blood pressure measurement as the reference standard for hypertension diagnosis.^28^ A precondition for clinical use of NA-AVR should therefore include an evaluation against ambulatory blood pressure measurement. A restricted age range was used (50–59 years) in this study. This was intended to reduce the effects of vascular changes that have been reported with age.^6–8 11^ A more comprehensive evaluation of the association of NA-AVR with hypertension would include an expanded age range. In this study, it was unknown if subjects had known hypertension or used antihypertensive medication. An additional limitation of the study is the lack of demographic characteristics to mitigate for cofounders, such as other conditions that may be simultaneously associated with high blood pressure and abnormal NA-AVR.

The clinical utility of the proposed system might be improved with further automation. For example, automatic arteriole–venule classification could be achieved by machine learning techniques.^29^ Further investigation over a larger age range and with ambulatory blood pressure measurement as a more accurate reference standard is now needed.
