# Supplementary material for: Association between hypertension and retinal vascular features in ultra-widefield fundus imaging
Source: Open Heart. 2020 Jan 8;7(1):e001124. doi: 10.1136/openhrt-2019-001124 (PMC6999694; doi:10.1136/openhrt-2019-001124)
Supplement: Supplementary data [file openhrt-2019-001124supp001.pdf]

Supplementary material

Table S1: Repeatability of successive measurements (repeat and original) for nasal-annular arteriole-venule ratio (NA-AVR) in: all eyes (All), left eyes (L) and right eyes (R). The arteriolar and venular components (µm) of NA-AVR are also shown. With Pearson's linear correlation coefficient (R^2), the 95% confidence interval (C) and 5% significance level (p). Significant p-values in **bold**.

		Pearson's linear correlation			Paired t-test			
Measure	Eye(s)	R^2	CI	p	Difference	CI	p	N
NA-AVR (unity)	All	0.82	0.74, 0.88	<0.005	-0.01	-0.02, 0.01	0.360	91
	L	0.77	0.61, 0.87	<0.005	-0.01	-0.03, 0.01	0.502	45
	R	0.86	0.76, 0.92	<0.005	-0.01	-0.02, 0.01	0.541	46
arterioles (µm)	All	0.91	0.87, 0.94	<0.005	0.0	-1.5, 1.5	0.107	91
	L	0.91	0.83, 0.95	<0.005	1.0	-0.3, 2.3	0.141	45
	R	0.92	0.86, 0.96	<0.005	0.4	-0.7, 1.5	0.455	46
venules (µm)	All	0.91	0.87, 0.94	<0.005	1.3	0.4, 2.2	**<0.005**	91
	L	0.94	0.89, 0.97	<0.005	1.9	0.6, 3.2	**0.006**	45
	R	0.88	0.79, 0.93	<0.005	1.0	-0.4, 2.3	0.180	46

Table S2: Area under receiver operator characteristic curve (AUC) for nasal-annular arteriole-venule ratio (NA-AVR) as a detector of hypertensive status in the study cohort. The arteriolar and venular components (µm) used to calculate NA-AVR are also given. AVR_5 uses the 5 widest arteriolar and venular segments, and AVR_{max} uses the widest arteriolar and venular segments. With 95% confidence intervals (CI) and 5% significance level (p). Significant p-values in **bold**.

		Left Eye		Right Eye		
Measure	Group	AUC	CI	AUC	CI	P
NA-AVR	All	0.73	0.68, 0.78	0.64	0.59, 0.70	**0.020**
	M	0.77	0.70, 0.83	0.59	0.51, 0.67	**<0.005**
	F	0.72	0.63, 0.78	0.68	0.58, 0.76	0.502
arterioles (µm)	All	0.70	0.65, 0.75	0.66	0.60, 0.71	0.307
	M	0.71	0.63, 0.77	0.62	0.54, 0.70	0.101
	F	0.71	0.63, 078	0.68	0.59, 0.75	0.616
venules (µm)	All	0.53	0.47, 0.59	0.52	0.47, 0.58	0.808
	M	0.49	0.40, 0.57	0.52	0.43, 0.60	0.611
	F	0.57	0.47, 0.65	0.55	0.46, 0.64	0.749
AVR_5	All	0.69	0.64, 0.74	0.63	0.57, 0.69	0.130
	M	0.74	0.66, 0.80	0.58	0.49, 0.65	**<0.005**
	F	0.66	0.58, 0.74	0.68	0.58, 0.76	0.742
AVR_{max}	All	0.68	0.63, 0.73	0.63	0.58, 0.69	0.209
	M	0.70	0.62, 0.77	0.61	0.53, 0.69	0.104
	F	0.67	0.58, 0.74	0.62	0.53, 0.70	0.416

Figure S1: Bland-Altman plots for measurement repeatability of the arteriolar and venular components (µm) of nasal-annular arteriole-venule ratio, showing the difference between successive measurements (repeat – original) on the y-axis and the average of the two measurements on the x-axis for: arterioles in (a) all eyes, (b) left eyes, (c) right eyes, and venules in (d) all eyes, (e) left eyes and (e) right eyes.
