# Supplementary figures and images for: Association between hypertension and retinal vascular features in ultra-widefield fundus imaging
Source: Open Heart. 2020 Jan 8;7(1):e001124. doi: 10.1136/openhrt-2019-001124 (PMC6999694; doi:10.1136/openhrt-2019-001124)

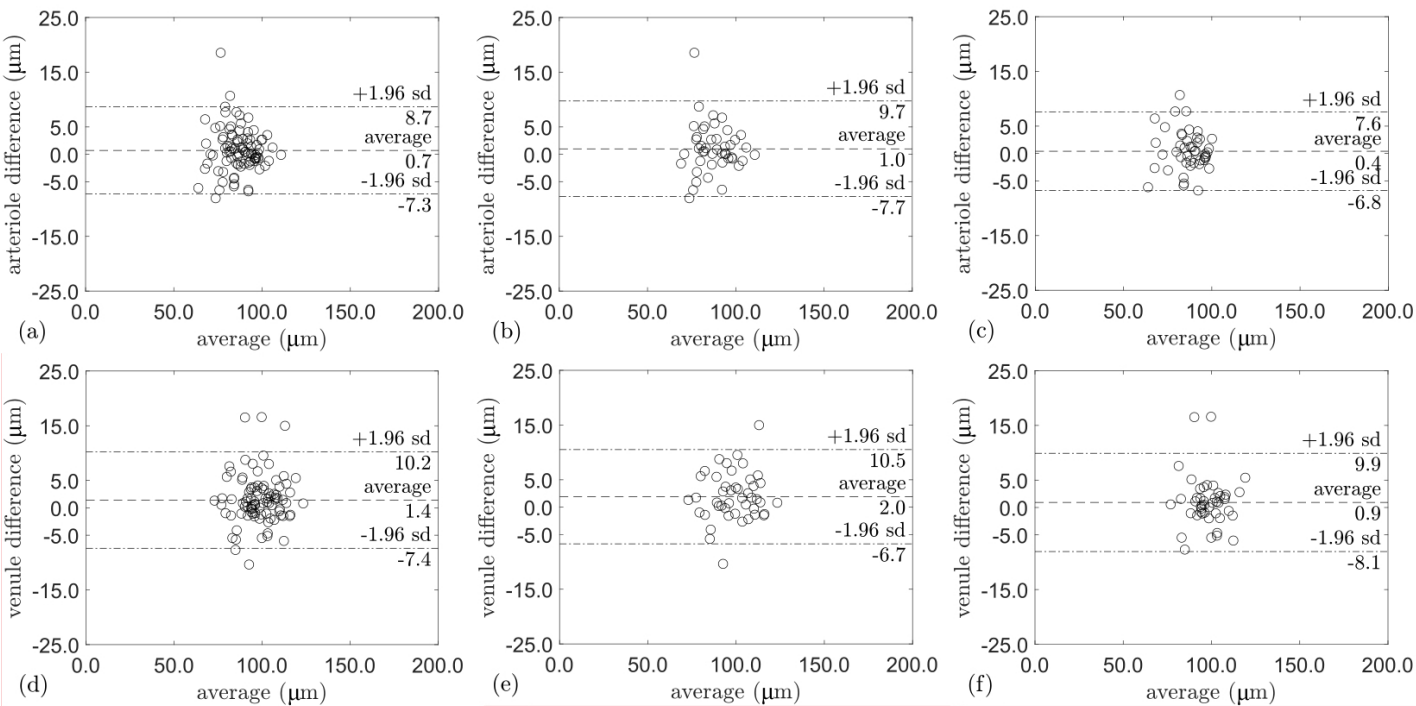

Supplement: Supplementary data [file openhrt-2019-001124supp002.pdf]
